# Effects of Tai Chi and Walking Exercises on Weight Loss, Metabolic Syndrome Parameters, and Bone Mineral Density: A Cluster Randomized Controlled Trial

**DOI:** 10.1155/2015/976123

**Published:** 2015-10-12

**Authors:** Stanley Sai-Chuen Hui, Yao Jie Xie, Jean Woo, Timothy Chi-Yui Kwok

**Affiliations:** ^1^Department of Sports Science and Physical Education, The Chinese University of Hong Kong, Shatin, New Territories, Hong Kong; ^2^Department of Medicine and Therapeutics, The Chinese University of Hong Kong, Shatin, New Territories, Hong Kong

## Abstract

Tai Chi and walking are both moderate-intensity physical activity (PA) that can be easily practiced in daily life. The objective of the study was to determine the effects of these two PAs on weight loss, metabolic syndrome parameters, and bone mineral density (BMD) in Chinese adults. We randomized 374 middle-aged subjects (45.8 ± 5.3 years) into 12-week training (45 minutes per day, 5 days per week) of Tai Chi (*n* = 124) or self-paced walking (*n* = 121) or control group (*n* = 129). On average, Tai Chi and walking groups lost 0.50 and 0.76 kg of body weight and 0.47 and 0.59 kg of fat mass after intervention, respectively. The between-group difference of waist circumference (WC) and fasting blood glucose (FBG) was −3.7 cm and −0.18 mmol/L for Tai Chi versus control and −4.1 cm and −0.22 mmol/L for walking versus control. No significant differences were observed regarding lean mass, blood pressure, triglycerides, total cholesterol, high-density and low-density lipoprotein cholesterol, and BMD compared to control. Change in lean mass, not fat mass or total weight loss, was significantly correlated to the change in BMD. Our results suggest that both of these two PAs can produce moderate weight loss and significantly improve the WC and FBG in Hong Kong Chinese adults, with no additional effects on BMD.

## 1. Introduction

Along with the increasing prevalence of obesity and sedentary lifestyles worldwide, the metabolic syndrome (MetSyn) has become a global public health problem [1–3]. The components of MetSyn are risk factors for many chronic conditions, including cardiovascular diseases, stroke, diabetes, and kidney disease, and result in the increased all-cause mortality [4–7]. More attention to lifestyle modifications for reducing obesity and promoting physical activity (PA) is thereby suggested [2]. In the Asia-Pacific region particularly greater China, rapid economic development coexists with the population aging [8]; the scientific and effective prevention of obesity and MetSyn in middle-aged people is thereby critical for healthy aging and health policy-making.

Evidences have shown that exercise is beneficial for preventing MetSyn [9–11]. The increased energy expenditure through exercise leads to weight loss and produces metabolic benefits [12]. A study conducted in America showed that moderate-intensity PA programs had significant effectiveness on reducing body fat and controlling for risk factors of MetSyn [11]. However, concern exists in the weight loss-related decline in bone mineral density (BMD) [12, 13]. Although there are many studies showing that exercise improved bone health in older adults [14], the contrary finding that exercise-induced weight loss caused a reduction in BMD has been reported [13], and some other studies demonstrated little or no effect on BMD [15–17]. The exercise type, intensity, and duration, as well as the target population, may lead to the discordance in study results. In addition, evidence has shown that lean mass exerts a greater effect on BMD than fat mass [18], thus investigating the changes of lean mass and fat mass during exercise which can help to better understand the effect of exercise-induced weight loss on BMD.

Tai Chi (also called Tai Chi Chuan or Taiji), a traditional Chinese mind-body exercise, is popular in Chinese population, particularly middle-aged and elderly people. Its physiological and psychosocial benefits on health outcomes have been well addressed [19, 20]. However, limited studies reported the effect of Tai Chi on all MetSyn parameters [21, 22]. We analyzed the data from a cluster randomized controlled trial (C-RCT) among generally healthy, inactive Hong Kong middle-aged people who practiced Tai Chi or self-paced brisk walking under the same frequency and duration for 12 weeks and kept the stable dietary intake in this period, with the purpose to see how much body weight would be reduced and to what extent would the MetSyn parameters be improved; if a significant weight loss was observed, what were the changes in fat and lean mass, whether this exercise-induced body mass loss had additional effect on BMD.

## 2. Method

### 2.1. Design and Participants

A 3-arm parallel-group C-RCT was designed. The whole research project has been briefly reported elsewhere [23] under the contractual obligation as a condition of funding approval. The experimental methods were carried out in accordance with relevant guidelines and regulations. All participants provided written informed consent. And all experimental protocols were approved by the Joint Chinese University of Hong Kong-New Territories East Cluster Clinical Research Ethics Committee. The study was registered at http://clinicaltrials.gov with the unique identifier number: NCT02163798.

Subjects were recruited from some large housing estates in the Shatin district of Hong Kong. These recruitment sites were classified as 9 geographic areas. Advertisements in flyers, surface mails, and bulletin boards were used for recruitment. The target subjects were aged 36 to 60. To screen eligible participants, all preliminary registered subjects were evaluated by health consultants using professional assessment form. Only inactive (no lifestyle physical activity or structured exercise experiences for at least 6 months [24]) persons were included. Those with cardiovascular and pulmonary diseases, neurological disorder, asthma, hearing and visual disabilities, communicable diseases, and musculoskeletal disorder were excluded.

### 2.2. Sample Size and Study Power

Based on the cluster randomized trial design, fixed three clusters per arm (9 geographic areas/3 arms) were determined in advance. The planned number of participants was 360, 120 in each arm, 40 in each cluster. Based on the conventional assumptions of two-sided 5% significance level and assuming the intracluster correlation was 0.01, according to the formula for fixed number of clusters each of fixed size by Donner and Klar [25, 26], we would have 80% power to detect a difference of 0.6 ± 1.1 kg in body weight, 11.4 ± 20.0 mg/cm^2^ in BMD, 3.5 ± 5.0 cm in waist circumference, 1.2 ± 2.0 mm Hg in systolic blood pressure (SBP), 0.20 ± 0.35 mmol/L in fasting blood glucose (FBG), 0.10 ± 0.17 mmol/L in total cholesterol, 0.05 ± 0.09 mmol/L in HDL-C, 0.02 ± 0.035 mmol/L in LDL-C, and 0.05 ± 0.09 mmol/L in triglycerides between the group means.

### 2.3. Randomization and Intervention

Participants recruited from one geographical area were considered as one cluster. The randomization was carried out at geographic area level for avoiding contamination. Nine clusters were then randomized to either Tai Chi, walking, or control arms, with the allocation ratio of 1 : 1. An independent statistician conducted the randomization using Excel to generate the allocation sequence. Another independent researcher critically carried out the allocation according to the sequence, to shield relevant investigators who might admit participants to the trial from knowing the upcoming assignments.

A modified 32-short form Yang-style Tai Chi Chuan was adopted in the Tai Chi group. Tai Chi integrates physical and spiritual elements to slowly and gently move* qi* (vital energy) throughout the body. Those rich with this* qi* feel wonderfully alive and strong. The Yang-style Tai Chi is the most popular and widely practiced Tai Chi style in the world. Its short form is typically done with slow, steady movements, which is a practical entry point for many beginners. Participants were required to practice 30 min per day, 5 days per week for 12 weeks. Within 1 week, the qualified Tai Chi Chuan instructors led 3 days of practices, and 2 other days were alloted for self-practice. Before and after the normal pratice, 10 min of warm-up stretching and 5 min of cool-down stretching were implemented, respectively. For the walking group, firstly, the qualified instructors gave a briefing session to the participants, to clarify the types of walking and demonstrated how to practice the standard self-paced brisk walking. Participants were advised to walk 30 min per day. They were encouraged to walk with their friends or families; the route of the walk could be a circle line or straight line depending on their preference. The warm-up and cool-down stretching, as well as the practice frequency, were similar as Tai Chi exercise. The actual implementations of practicing Tai Chi and walking were recorded by an exercise log. For those allocated to control group, they were told that they would be provided two sessions of free health and fitness assessment with an interval of three months (12 weeks). No additional exercise traning was provided.

### 2.4. Data Collection

Individual information indluding sociodemographic characteristics (age, sex, residential area, and housing estate), medical history, and medication were obtained at baseline by face-to-face interviews. Participants were also required to provide a 1-week diet food frequency data, which were collected by the Food Frequency Questionnaire (FFQ). Dietary nutrients (energy, protein, and total fat) were then calculated. The MetSyn parameters include waist circumference, blood pressure (BP), FBG, total cholesterol, HDL-C, LDL-C, and triglycerides [2]. These data, together with the dietary intake information and BMD, were collected twice: at baseline and upon completion of the 12-week program. The independent assessors who collected outcome data were unaware of the assigned intervention until the end of the trial. An external private clinic was delegated to analyze the blood samples. All staffs in this clinic did not know the assigned intervention.

#### 2.4.1. Measurement of Anthropometrics and BP

Weight, height, and waist circumference were measured to the nearest 0.1 kilogram or centimeter based on a standard protocol. BMI was then calculated (BMI = weight (kg)/height (m^2^)). All measurements were conducted twice by the trained research assistant; the average value was used. Waist circumference was measured by a standardized Gulick tape. To avoid the contraction of abdominal muscles, the measurement was recorded after the participant exhaled gently in normal breathing. The measurement position was at a level midway between the lower rib margin and the iliac crest. Obesity and central obesity were defined as BMI ≥ 25 kg/m^2^ and waist circumference ≥ 80 cm for women and ≥ 90 cm for men, respectively [27]. BP was measured to the nearest 0.1 millimeter of mercury (mm Hg). A corrected mercury sphygmomanometer and an appropriately sized cuff were used. Measurements were taken after at least ten minutes of seated rest. The mean of two times measurements was computed. Participants who were currently taking antihypertension medicine were asked to not take it before coming to the laboratory for body measurements. Hypertension was defined as either a SBP of ≥ 140 mm Hg or a diastolic blood pressure (DBP) of ≥ 90 mm Hg [28].

#### 2.4.2. Measurement of Body Mass and Bone Mineral Density

Body mass and BMD were measured by Hologic QDR-2000 dual-energy X-ray densitometer (Hologic, Bedford, MA, US) at baseline and 12 weeks. The machine provides values for lean mass, fat mass, BMD, and total body water assuming that water constitutes 73.2% of lean mass. The BMD were measured at the hip (femoral neck, intertrochanteric area, and the total hip), the spine (L1–L4), and the total body. The values from total body were used in current analysis. The stability of the machine and the long-term precision of the measurement have been identified acceptable where the coefficient of variance (CV%) was less than 0.42% in a previous study using the same machine [29].

#### 2.4.3. Blood Samples and Biochemical Parameters

Overnight fasting (10–12 h) venous blood samples were obtained at 8–10 am for the measurement of glucose and lipid concentrations. Blood withdrawal was conducted by professional nurses. Participants who had acute inflammation or taking anti-inflammation drugs (i.e., aspirin or antibiotics) at the time of completing 12-week program were required to postpone 1 week for blood withdrawal. The blood samples were centrifuged at 3000 ×g for 15 min under condition of 4°C. The serum was isolated within 2 h after collection and divided into several aliquots and stored at −85°C until analysis. Serum was measured by enzymatic methods and serum ACE (Gcell, Beijing Strong Biotechnologies, Inc.) by colorimetric assay. The FBG, total cholesterol, high-density lipoprotein cholesterol (HDL-C), and triglycerides were measured with a modified hexokinase enzymatic method (7020 clinical analyzer, Hitachi, Tokyo, Japan).

### 2.5. Statistical Analysis

Statistical analysis was performed by SPSS 20.0 (SPSS Institute) software. Data were analyzed according to an intention-to-treat (ITT) principle. All participants who attended baseline assessment were involved in the ITT. Baseline differences between three groups were compared by one-way ANOVA and Pearson chi-square test for continuous and categorical variables, respectively. Post hoc procedures were further conducted for the pairwise comparison. One-way analysis of covariance (ANCOVA) was used to compare the mean changes of outcomes from baseline to after intervention (12-week) between three groups. To compare the between-group differences in mean change from baseline to 12-week, repeated measures of ANCOVA were implemented. The time × group interaction effects between intervention group and control group, as well as between the two intervention groups, were examined. The variables that were significantly different between groups at baseline were adjusted as covariates in the ANCOVA models. Bivariate correlations between main outcomes at baseline, as well as the intentional weight (and body mass) loss, and changes in BMD and MetSyn parameters at 12 weeks were examined by Spearman's correlation test. *P* value < 0.05 was considered as statistically significant.

## 3. Results

A total of 398 subjects from 9 locations were successfully enrolled in the study. 24 subjects (3 in the Tai Chi group, 2 in the walking group, and 19 in the control group) quit after randomization because they were unwilling to participate in the allocated group. Finally 374 subjects attended baseline assessment. Detailed study flow and intervention allocating were indicated in the Supplementary Figure 1 (see Supplementary Material available online at http://dx.doi.org/10.1155/2015/976123). The number of valid participants was 129, 121, and 124 in Tai Chi, walking, and control group, respectively. After 12 weeks follow-up, 21 (5.6%) participants withdrew with eight in the Tai Chi group, ten in the walking group, and three in the control group. The retention rates in the three groups were all higher than 90%. No significant differences were observed in the baseline characteristics between participants who completed the entire study and those who were lost to follow-up (all *P* > 0.05).

### 3.1. Baseline Characteristics

The mean age of participants in Tai Chi, walking, and cotrol groups was 45.9 ± 5.2, 46.6 ± 4.9, and 44.9 ± 5.6 years, respectively. The difference was statistically significant (*P* = 0.034). Other baseline characteristics are shown in Table 1. No significant differences were observed among the three groups in terms of age and gender proportions, dietary intake, proportions of obesity, and hypertension (all *P* > 0.05). Nevertheless, the proportion of central adiposity was higher in Tai Chi and walking groups than in the control group (34.1%, 34.7%, and 17.7%, *P* = 0.004).

### 3.2. Intervention Effects on Weight Loss, MetSyn Parameters, and BMD

Table 2 shows the values of all outcomes from basline to 12 weeks, as well as the mean changes between the two time meaures; Table 3 shows the pairwise comparisons of between-group difference at 12 weeks. At baseline, participants in the Tai Chi and walking groups were more likely to have larger waist circumferences and higher FBG than those in the control group (all *P* < 0.01, Table 2). Age, baseline waist circumference, baseline fasting glucose, and HDL-C were adjusted as covariates in corresponding ANCOVA models because of their imbalance among groups at baseline. The dietary intake was not significantly different between three groups at 12 weeks (*P* > 0.05, data not shown).

From baseline to 12 weeks, on average, Tai Chi and walking groups significantly lost 0.50 and 0.76 kg of body weight and 0.47 and 0.59 kg of fat mass, respectively (all *P* < 0.01, Table 2). The between-group difference of weight loss was −0.60 kg for Tai Chi versus control (95% confidence interval (CI): −0.94 to −0.25) and −0.85 kg for walking versus control (95% CI: −1.20 to −0.50) (both *P* < 0.001, Table 3). No significant changes of lean mass and BMD in both intervention groups and control group were observed (all *P* > 0.05).

**Figure 1:**
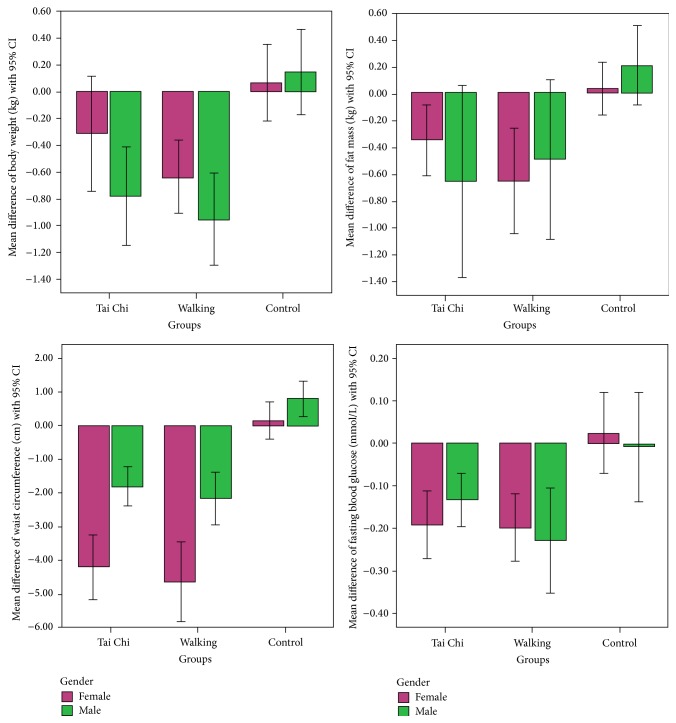
Mean differences of body weight, total fat mass, waist circumference, and fasting blood glucose from baseline to 12 weeks by male and female in three groups.

Participants in two intervention groups also had significantly greater decreases in waist circumference and FBG than those in the control group (Table 2). The mean decrease of waist circumference was 3.3 cm in both intervention groups (95% CI: −3.9 to −2.6 for Tai Chi group and −4.5 to −2.9 for walking group). The between-group difference was −3.7 cm for Tai Chi versus control (95% CI: −4.4 to −2.9) and −4.1 cm for walking versus control (95% CI: −5.0 to −3.2) (both *P* < 0.001, Table 3). For FBG, participants in the Tai Chi and walking groups had a mean decrease of 0.17 mmol/L (95% CI: −0.22 to −0.11) and 0.21 mmol/L (95% CI: −0.28 to −0.14), respectively (Table 2). It showed significant reductions than the control group (0.01 mmol/L, 95% CI: −0.06 to 0.09, *P* < 0.001). The mean between-group difference was −0.18 mmol/L for Tai Chi versus control (95% CI: −0.27 to −0.09) and −0.22 mmol/L for walking versus control (95% CI: −0.31 to −0.13) (both *P* < 0.001, Table 3). The BP also showed notable reductions in both intervention groups. For SBP, mean change was −2.6 mm Hg and −2.4 mm Hg for Tai Chi and walking groups, respectively. However, the changes were not significantly different when compared with control (both *P* > 0.05, Table 2). The mean between-group differences were also small and not significant (all *P* > 0.05). Similar situation was found in DBP. For all other outcomes, including total cholesterol, HDL-C, LDL-C, and triglycerides, both Tai Chi and walking training showed no significant effects when compared with control (all *P* > 0.05, Tables 2 and 3). When we compared the effects between two intervention groups, all outcomes showed no significant differences at 12 weeks (all *P* > 0.05, Table 3).  Figure 1 shows the changes in body weight, fat mass, waist circumference, and FBG in three groups by male and female seperately. Females had significantly higher reduction in waist circumfernce than males after intervention (*P* < 0.05).

### 3.3. Relationships of Weight Loss with BMD and MetSyn Parameters

The correlation coefficients between changes in body weight, fat mass, lean mass, waist circumference, BMD, and other MetSyn parameters at 12 weeks in the two intervention groups were shown in Table 4. Loss in body weight and fat mass both positively correlated with the decrease in waist circumference (*r* = 0.38 and 0.19, resp., both *P* < 0.01); the changes in waist circumference also correlated with the changes in SBP and FBG (*r* = 0.15 and 0.23, both *P* < 0.05). For the BMD, however, only the changes in lean mass showed significant correlation with it (*r* = 0.41, *P* < 0.01). Correlation analysis of these outcomes at baseline showed similar results (in all participants, data not shown).

## 4. Discussion

Results from this exploratory study provide novel information about the effects of 12-week Tai Chi and brisk walking trainings on weight loss, BMD, and MetSyn parameters in middle-aged Hong Kong adults. We found that these two moderate-intensity, short-term PA programs both slightly reduced the body weight and fat mass and had significant improvements on waist circumference and FBG. These two interventions showed no apparent effect on BMD. Furthermore, we found that BMD only correlated with the lean mass; the exercise-induced weight loss, particular loss in fat mass, had no significant associations with the changes in BMD.

The effect of Tai Chi on weight loss is inconsistent in the literature. A study [30] conducted in postmenopausal women showed that the body weight and fat mass in dynapenic women reduced 1.5 kg and 1.1 kg, respectively, whereas no change was found in nondynapenic women. Another study among transitionally frail older adults showed a mean decrease of 1.49 kg in body weight after 48 weeks training of Tai Chi [31]. Compared with these studies, our 12-week trial obtained a smaller but still significant reduction in body weight and fat mass. However, some previous studies conducted in elderly Chinese subjects [32] and in old women with osteoarthritis [33] showed no significant change in body weight/BMI. These heterogeneous findings indicate that the training time, the frequency, and duration of exercise, as well as the target population, are all important in detecting significant clinically relevant effect.

Exercise is a well-known lifestyle description for managing type 2 diabetes or impaired glucose tolerance. However, whether Tai Chi has any benefit on glucose control is conflicted in the literature [34]. Some studies have shown their benefits on blood glucose in diabetic patients [35, 36], whereas other studies demonstrated limited effect or no effect [22, 32, 37]. Of note, all these studies had relatively small sample size or non-RCT design. Our study provides evidence that Tai Chi and walking can both significantly decrease FBG in adults. Regarding total cholesterol, HDL-C, LDL-C, and triglycerides, no significant changes were observed in the study. For the BP, an apparent within-group pre-postchange was found in Tai Chi and walking participants, but this effect was limited because no significant difference was observed compared to control. Evidence has shown that reductions in BP appear to be more pronounced in hypertensive subjects [38–40]. Smaller effect size observed in our study may be due to the fact that most participants were normotensive subjects. Whether Tai Chi or walking can serve as the nonpharmacologic adjuncts to prevent hypertension may need long-term observation.

No significant change in BMD was observed in our study. A study in Hong Kong elderly people indicated that short-term Tai Chi exercise (e.g., 12 weeks) may not provide sufficient training stimulation in improving bone health [41]. Other studies concluded that Tai Chi can act as a protective factor for bone loss in old women [42, 43]. Similar debates exist in walking [14, 15, 44, 45]. It may need a more definitive, long-term trial to examine whether Tai Chi or walking have protective effect on bone loss. In our study, we further identified that the moderate exercise-induced weight loss had no significant association with reductions in BMD; it is comparable to a previous study [16]. This study also found no impact of exercise-induced weight loss on BMD when similarly the caloric intake was kept stable for the exercise group. Furthermore, we found that only the change in lean mass was associated with the change in BMD; the reduction in fat mass did not influence the BMD. The finding was consistent with previous studies [18]. On the other hand, we found a significant decrease in waist circumference; it was positively correlated with the reductions in body weight and fat mass. All these findings imply that Tai Chi and walking exercises may both be suitable for designing weight loss therapy program or obesity management program in middle-aged adults, because they have no additional adverse effect on BMD and can reduce fat mass and improve waist circumference.

Our study presents several limitations. First, predetermined number of clusters per arm (9 clusters/3 arms) in our cluster RCT led to imbalance in some variables. Participants in the control group seemed to have better waist circumference and FGB than those in intervention groups at baseline. However, we suggest that the improvement effects in these two variables are unlikely due to worse shape in intervention groups, because the significant within-group improvement was also observed. Nevertheless, the effect sizes might be slightly overestimated. Second, double-blind study design was not available due to the difficulty of administration and operation. Participants in the intervention groups might have higher expectations of the treatment results. This awareness of intervention assignments might introduce some bias into the results. Third, 12 weeks of intervention may not be long enough to observe significant improvement on certain health outcomes; thus, a longer-term follow-up study is suggested.

In summary, our study was the first to comprehensively examine the effects of a type of mind-body exercise and a simple physical exercise on the MetSyn parameters, weight loss, and BMD with a certain large sample size and using a cluster RCT design. We found that these two exercise interventions moderately reduced the body weight and fat mass and improved the waist circumference and FBG, and the exercise-induced weight loss did not impact the BMD. We suggest that Tai Chi and walking are both feasible and promising daily moderate PA for middle-aged adults. Findings from this study provide referable information for current public health initiatives to health aging and future community-based moderate PA and lifestyle intervention programs.

## Supplementary Material

Supplementary Figure 1: Flow diagram of Tai Chi/walking cluster randomized control trial. The number of subjects at the stages of enrollment, randomization, baseline assessment, and analysis were shown in this flow diagram. The detailed intervention allocating and follow up were also demonstrated.

## Figures and Tables

**Table 1:** Baseline characteristics of participants and comparisons among groups.

	Tai Chi group	Walking group	Control group	*P* value^a^
Geographic areas (*n*)	3	3	3	
Participants (*n*)	129	121	124	
Age group, *n* (%)				0.068
36–40	18 (14.0)	14 (11.6)	30 (24.2)	
41–50	89 (69.0)	82 (67.8)	71 (57.3)	
51–60	22 (17.1)	25 (20.7)	23 (18.5)	
Gender, *n* (%)				0.863
Female	77 (59.4)	75 (62.0)	78 (62.9)	
Male	52 (40.3)	46 (38.0)	46 (37.1)	
Dietary intake				
Energy, kCal/day, mean (SD)	2094.9 (838.8)	2159.4 (1135.1)	2216.2 (898.6)	0.608
Protein, g/day, mean (SD)	93.7 (20.4)	100.4 (28.1)	88.1 (38.7)	0.494
Fat, g/day, mean (SD)	88.5 (25.5)	84.6 (29.1)	85.9 (25.1)	0.909
Obesity, BMI ≥ 25 kg/m^2^, *n* (%)				0.446
Yes	41 (31.8)	32 (26.4)	31 (25.0)	
No	88 (68.2)	89 (73.6)	93 (75.0)	
Central adiposity, waist circumference ≥ 80 cm, *n* (%)				0.004
Yes	44 (34.1)	42 (34.7)	22 (17.7)	
No	85 (65.9)	79 (65.3)	102 (82.3)	
Hypertension, *n* (%)				0.180
Yes	13 (10.1)	20 (16.5)	12 (9.7)	
No	116 (89.9)	101 (83.5)	112 (90.3)	
Metabolic syndrome, *n* (%)^b^				0.662
Yes	16 (12.4)	13 (10.7)	11 (8.9)	
No	113 (87.6)	108 (89.3)	113 (91.1)	
Presence of any 1 of 5 risk factors of metabolic syndrome, *n* (%)				0.064
Yes	83 (64.3)	72 (59.5)	62 (50.0)	
No	46 (35.7)	49 (40.5)	62 (50.0)	
Presence of any 2 of 5 risk factors of metabolic syndrome, *n* (%)				0.142
Yes	44 (34.1)	30 (24.8)	30 (24.2)	
No	85 (65.9)	91 (75.2)	94 (75.8)	

^a^ *P* values generated from one-way ANOVA or Pearson chi-square test where appropriate.

^b^The presence of any 3 of 5 risk factors constitutes a diagnosis of metabolic syndrome: (1) central adiposity; (2) triglycerides ≥ 1.7 mmol/L; (3) high-density lipoprotein cholesterol < 1.0 mmol/L in males; <1.3 mmol/L in females; (4) systolic BP ≥ 130 and/or diastolic BP ≥ 85 mm Hg; (5) blood fasting glucose ≥ 100 mg/dL.

**Table 2:** Changes of weight, body mass, bone mineral density, and metabolic syndrome parameters from baseline to 12 weeks in each group.

Measure^a^	Tai Chi group (*n* = 129)	Walking group (*n* = 121)	Control group (*n* = 124)	*P* value^b^
Weight (kg)				
Baseline	61.1 (11.2)	61.1 (11.0)	59.9 (11.0)	0.661
12-week	60.6 (11.0)	60.3 (10.8)	60.0 (11.0)	0.933
Mean change from baseline	−0.50 (−0.80 to −0.21)	−0.76 (−0.97 to −0.55)	0.1 (−0.12 to 0.31)	<0.001
Total body fat mass (kg)				
Baseline	18.6 (49.8)	18.8 (52.8)	18.1 (60.9)	0.580
12-week	18.1 (51.9)	18.2 (49.7)	18.2 (60.7)	0.980
Mean change from baseline	−0.47 (−0.79 to −0.15)	−0.59 (−0.92 to −0.26)	0.09 (−0.07 to 0.26)	0.002
Total body lean mass (kg)				
Baseline	39.4 (90.9)	39.5 (92.1)	39.2 (85.3)	0.968
12-week	39.4 (92.4)	39.3 (92.5)	39.1 (86.1)	0.987
Mean change from baseline	−0.03 (−1.01 to 0.96)	−0.11 (−0.64 to 0.42)	−0.10 (−0.24 to 0.04)	0.714
Waist circumference (cm)				
Baseline	80.9 (8.8)^*∗∗*^	81.1 (9.4)^*∗∗*^	76.1 (9.8)	<0.001
12-week	77.7 (9.1)	77.4 (9.6)	76.5 (9.8)	0.585
Mean change from baseline	−3.3 (−3.9 to −2.6)	−3.3 (−4.5 to −2.9)	0.4 (−0.0 to 0.8)	<0.001
Systolic blood pressure (mm Hg)				
Baseline	114.8 (15.4)	112.9 (16.0)	113.3 (14.3)	0.561
12-week	112.3 (14.6)	110.4 (14.3)	112.0 (13.9)	0.560
Mean change from baseline	−2.6 (−4.3 to −0.8)	−2.4 (−4.2 to −0.7)	−1.4 (−3.0 to 0.2)	0.509
Diastolic blood pressure (mm Hg)				
Baseline	74.4 (11.7)	74.9 (13.2)	75.3 (11.0)	0.929
12-week	72.9 (11.5)	71.7 (12.9)	72.3 (11.9)	0.704
Mean change from baseline	−1.8 (−3.2 to −0.3)	−3.2 (−4.7 to −1.7)	−2.9 (−4.4 to −1.5)	0.397
Fasting blood glucose (mmol/L)				
Baseline	4.8 (0.5)^*∗∗*^	4.8 (0.4)^*∗∗*^	4.5 (0.4)	<0.001
12-week	4.6 (0.5)	4.6 (0.5)	4.5 (0.5)	0.355
Mean change from baseline	−0.17 (−0.22 to −0.11)	−0.21 (−0.28 to −0.14)	0.01 (−0.06 to 0.09)	<0.001
Total cholesterol (mmol/L)				
Baseline	5.1 (0.9)	5.1 (0.9)	5.0 (0.9)	0.363
12-week	5.1 (1.0)	4.9 (0.9)	4.8 (0.8)	0.064
Mean change from baseline	−0.07 (−0.16 to 0.03)	−0.13 (−0.23 to −0.03)	−0.18 (−0.27 to −0.09)	0.235
HDL-C (mmol/L)				
Baseline	1.5 (0.4)	1.7 (0.5)^*∗*^	1.5 (0.4)	0.035
12-week	1.5 (0.4)	1.6 (0.5)	1.4 (0.4)	0.033
Mean change from baseline	−0.03 (−0.08 to 0.03)	−0.09 (−0.14 to −0.05)	−0.10 (−0.15 to −0.06)	0.087
LDL-C (mmol/L)				
Baseline	3.0 (0.8)	2.9 (0.8)	2.9 (0.8)	0.645
12-week	3.0 (0.9)	2.9 (0.8)	2.9 (0.7)	0.528
Mean change from baseline	−0.01 (−0.11 to 0.09)	−0.03 (−0.11 to 0.06)	−0.03 (−0.12 to 0.06)	0.932
Triglycerides (mmol/L)				
Baseline	1.4 (1.0)	1.2 (0.7)	1.3 (1.0)	0.121
12-week	1.4 (0.9)	1.1 (0.6)	1.2 (0.9)	0.017
Mean change from baseline	0.01 (−0.08 to 0.09)	−0.05 (−0.13 to 0.03)	−0.10 (−0.20 to 0.00)	0.204
Bone mineral density (mg/cm^2^)				
Baseline	1037.3 (79.6)	1039.3 (85.0)	1034.1 (82.3)	0.887
12-week	1036.9 (79.1)	1037.6 (85.0)	1033.8 (83.5)	0.930
Mean change from baseline	−0.39 (−6.57 to 5.80)	−1.65 (−5.16 to 1.85)	−0.33 (−2.43 to 1.78)	0.480

HDL-C: high-density lipoprotein cholesterol; LDL-C: low-density lipoprotein cholesterol.

^a^Values were presented as mean (SD) for 12-week measurement and mean (95% CI) for mean change from baseline; mean change from baseline = 12-week evaluation − baseline evaluation.

^b^One-way ANOVA was used to compare the mean difference at baseline and at 12 weeks between groups; univariate ANCOVA was used to compare the mean change difference between groups; variables with significant different between groups at baseline were adjusted as covariates.

^*∗*^Post hoc comparison was used; differences are significant (*P* < 0.05) between intervention and control group.

^*∗∗*^Post hoc comparison was used; differences are significant (*P* < 0.01) between intervention and control group.

**Table 3:** Between-group differences of body weight, body mass, metabolic syndrome parameters, and bone mineral density after intervention.

Measure	Tai Chi versus control^a^		Walking versus control^a^		Tai Chi versus walking^a^	
	Between-group difference (95% CI)	*P* value	Between-group difference (95% CI)	*P* value	Between-group difference (95% CI)	*P* value
Weight (kg)	−0.60 (−0.94 to −0.25)	0.008	−0.85 (−1.20 to −0.50)	<0.001	0.26 (−0.09 to 0.60)	0.164
Total body fat mass (kg)	−0.56 (−0.96 to −0.17)	0.004	−0.68 (−1.08 to −0.28)	<0.001	0.12 (−0.28 to 0.51)	0.353
Total body lean mass (kg)	0.08 (−0.86 to 1.01)	0.926	−0.01 (−0.96 to 0.94)	0.858	0.08 (−0.85 to 1.02)	0.878
Waist circumference (cm)	−3.7 (−4.4 to −2.9)	<0.001	−4.1 (−5.0 to −3.2)	<0.001	0.5 (−0.6 to 1.5)	0.489
Systolic blood pressure (mm Hg)	−1.2 (−3.6 to 1.1)	0.311	−1.1 (−3.5 to 1.3)	0.283	0.4 (−0.5 to 1.3)	0.824
Diastolic blood pressure (mm Hg)	1.2 (−0.9 to 3.2)	0.568	−0.3 (−2.4 to 1.8)	0.340	1.4 (−0.7 to 3.5)	0.201
Fasting blood glucose (mmol/L)	−0.18 (−0.27 to −0.09)	<0.001	−0.22 (−0.31 to −0.13)	<0.001	0.04 (−0.05 to 0.13)	0.366
Total cholesterol (mmol/L)	0.11 (−0.02 to 0.24)	0.095	0.05 (−0.09 to 0.18)	0.269	0.06 (−0.07 to 0.20)	0.453
HDL-C (mmol/L)	0.07 (0.01 to 0.14)	0.330	0.01 (−0.06 to 0.08)	0.705	0.06 (−0.004 to 0.13)	0.060
LDL-C (mmol/L)	0.02 (−0.11 to 0.15)	0.850	0.002 (−0.13 to 0.14)	0.882	0.02 (−0.11 to 0.15)	0.708
Triglycerides (mmol/L)	0.10 (−0.02 to 0.23)	0.085	0.05 (−0.17 to 0.08)	0.693	0.06 (−0.06 to 0.18)	0.265
Bone mineral density (mg/cm^2^)	−0.06 (−6.16 to 6.03)	0.477	1.33 (−7.52 to 4.86)	0.295	1.27 (−4.85 to 7.39)	0.573

HDL-C: high-density lipoprotein cholesterol; LDL-C: low-density lipoprotein cholesterol.

^a^ *P* values were calculated for the time × group interaction effects from baseline to 3 months between groups by repeated ANCOVA; variables with significantly different between groups at baseline were adjusted as covariates.

**Table 4:** Correlation coefficients of weight loss, fat mass, lean mass, waist circumference with BMD, and metabolic syndrome parameters^a^.

	Δ BMD	Δ waist circumference	Δ SBP	Δ DBP	Δ FBG	Δ TC	Δ HDL-C	Δ LDL-C	Δ triglycerides
Weight loss (Δ body weight)	−0.05	0.38^*∗∗*^	0.13^*∗*^	0.04	0.03	0.09	−0.01	0.05	0.20^*∗∗*^
Δ fat mass	0.05	0.19^*∗∗*^	0.03	0.06	0.07	0.12^*∗*^	0.05	0.11^*∗*^	0.04
Δ lean mass	0.41^*∗∗*^	0.09	−0.08	−0.06	−0.01	−0.02	−0.05	−0.04	0.05
Δ waist circumference	0.01	1.00	0.15^*∗∗*^	0.03	0.23^*∗∗*^	0.00	0.00	0.00	0.01

BMD: bone mineral density; SBP: systolic blood pressure; DBP: diastolic blood pressure; FBG: fasting blood glucose; TC: total cholesterol; HDL-C: high-density lipoprotein cholesterol; LDL-C: low-density lipoprotein cholesterol.

^a^Correlation analyses were conducted between pre-post differences (Δ) of body weight, fat mass, lean mass, waist circumference and pre-post differences (Δ) of bone mineral density (BMD) and other metabolic syndrome parameters. Analysis only included cases in Tai Chi and walking groups. Pre-Post difference (Δ) = post value − pre value.

^*∗∗*^Correlation is significant at the 0.01 level (2-tailed).

^*∗*^Correlation is significant at the 0.05 level (2-tailed).
