# Supplementary figures and images for: Effects of Tai Chi and Walking Exercises on Weight Loss, Metabolic Syndrome Parameters, and Bone Mineral Density: A Cluster Randomized Controlled Trial
Source: Evid Based Complement Alternat Med. 2015 Oct 12;2015:976123. doi: 10.1155/2015/976123 (PMC4620402; doi:10.1155/2015/976123)

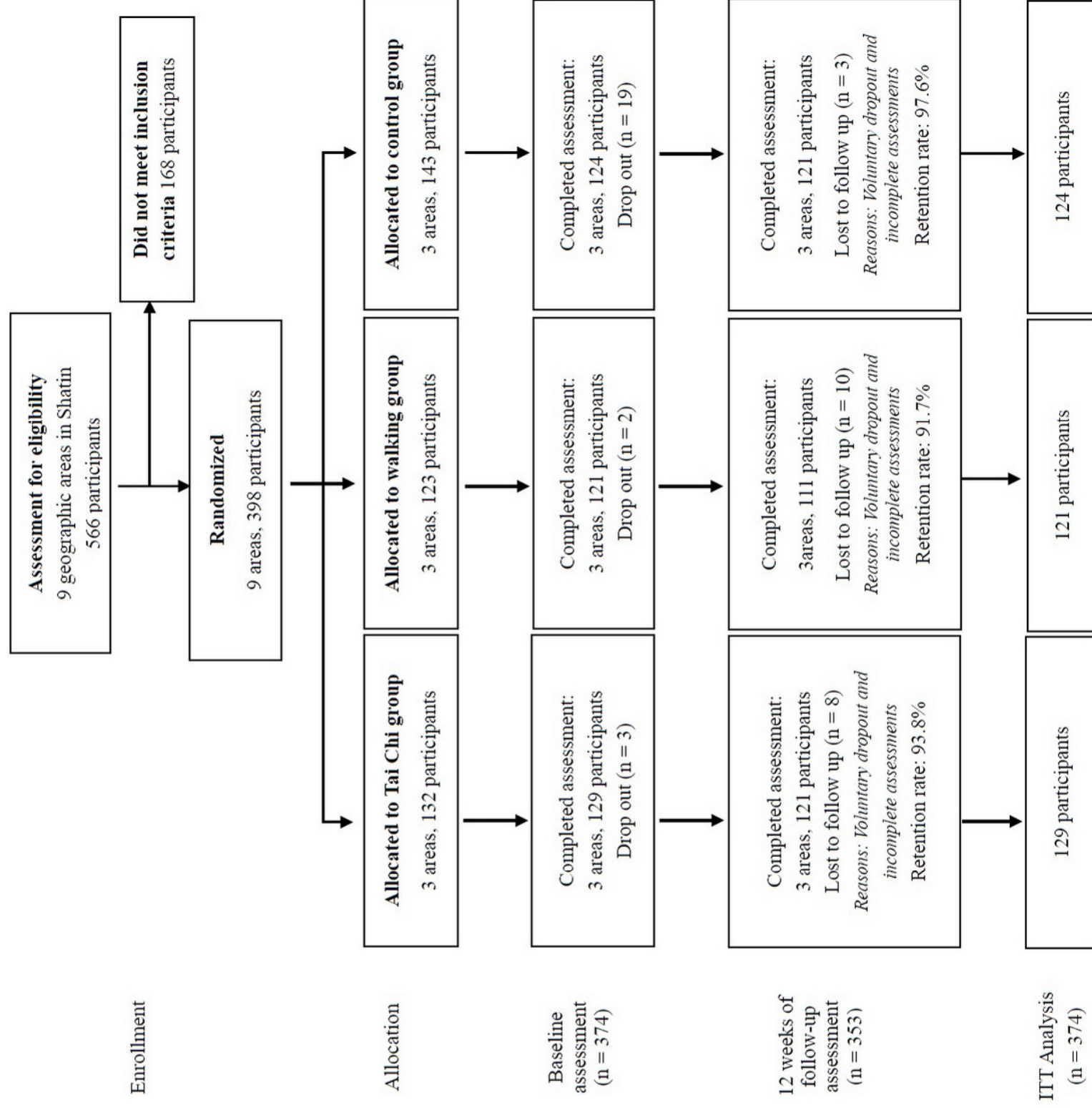

Supplement: Supplementary file 1 — Supplementary Figure 1: Flow diagram of Tai Chi/walking cluster randomized control trial. The number of subjects at the stages of enrollment, randomization, baseline assessment, and analysis were shown in this flow diagram. The detailed intervention allocating and follow up were also demonstrated. [file 976123.f1.pdf]
